# The Forty-First Annual Commencement of the Balto. College of Dental Surgery

**Published:** 1881-03

**Authors:** 


### EDITORIAL, ETC.

*The Forty-first Annual Commencement of the Baltimore College of Dental Surgery* was held March 2nd, 1881, at 2 P. M. at the Academy of Music, Baltimore City. A large and brilliant audience filled the spacious auditorium, and everything passed off successfully and pleasantly. The Fifth Regiment Band, Director Itzel, furnished enlivening music. After prayer by the Rev. William Kirkus, D. D., Rector of St. Michael and all Angels' Church, the names of the graduates were announced and the degree of "Doctor of Dental Surgery" conferred by Prof. F. J. S. Gorgas, Dean, on the following gentlemen:

| | |
|---|---|
| Samuel B. Adair, | *Georgia.* |
| Roderick Ashton Barrick, | *Maryland.* |
| Ellis B. Bliss, | *Dist. Columbia.* |
| Ludwig Brandt, | *Germany.* |
| Benijah S. Byrnes, | *Mississippi.* |
| James Valentine Calver, | *Dist. Columbia.* |
| George White Carmen, | *New Jersey.* |
| Myron A. Carman, | *New York.* |
| Leonard T. Caughy, | *Maryland.* |
| Thomas E. Craddock, | *Virginia.* |
| Robert Byron Cummins, | *Pennsylvania.* |

| | |
|---|---|
| Edward R. De Normandie, | *Pennsylvania.* |
| Benjamin Hale Douglass, | *Massachusetts.* |
| Byrd Page Dunnavant, | *Virginia.* |
| Frank O. Eilenberger, | *Illinois.* |
| Charles Marshall Emmart, | *Maryland.* |
| Benjamin Flannigain, | *Maryland.* |
| William G. Foster, | *Maryland.* |
| Theodorick Thomas Frazier, | *North Carolina.* |
| Laurence De Lancy Gorgas, | *Maryland.* |
| James H. Grant, | *Texas.* |
| Charles Church Harris, | *Maryland.* |
| Howard William Hoopes, | *Maryland.* |
| James A. Hurdle, | *North Carolina.* |
| John G. Keller, | *Georgia.* |
| Daniel O. Knight, | *Dist. Columbia.* |
| Henry D. Kurtz, | *New York.* |
| Frank Adolph Lee, | *Virginia.* |
| Myron M. Maine, | *Connecticut.* |
| Lemuel Estes Meador, | *South Carolina.* |
| Frank S. Milbury, | *New Brunswick.* |
| Robert W. Morgan, | *Virginia.* |
| Edgar P. Parsons, | *West Virginia.* |
| H. Spencer Pitts, | *Virginia.* |
| Edgar R. Rust, | *Virginia.* |
| P. Henry Salles, | *Louisiana.* |
| Frank Morris Seebold, | *Pennsylvania.* |
| James J. Seigler, | *South Carolina.* |
| Damian Silva, | *Cuba.* |
| B. Holly Smith, Jr., | *Maryland.* |
| Edwin Byron Smith, | *Pennsylvania.* |
| Frank Alexander Speck, | *Tennessee.* |
| Charles Lowndes Steel, | *Virginia.* |
| Robert Wilson Sterling, | *South Africa.* |
| Caleb Anderson Thompson, | *Virginia.* |
| Wesley Fletcher Tigner, | *Georgia.* |
| William Townes, Jr. | *Virginia.* |
| John Charles Wachter, | *Maryland.* |
| Effingham Wagner, | *Alabama.* |
| John Henry Walker, | *Louisiana.* |
| William Austin White, | *New York.* |
| W. Cuttino Wilbur, | *South Carolina.* |
| E. Powell Wright, | *Virginia.* |

Many floral offerings, costly, beautiful and unique in design were presented to the graduates by their lady friends, who composed a large part of the audience.

The Valedictory Address was then delivered by the Rev. William Kirkus, D. D., in the able and attractive manner so characteristic of this well known and highly esteemed clergyman; and which we present to our readers in the present number of the Journal.

The presentation of the Collegiate Prizes was then made by Dr. M. W. Foster, President of the Board of Visitors, as follows:

The First Prize.—A Case of Dental Instruments, to Laurence De Lancy Gorgas, of Maryland, for the highest number of votes at the final examinations on all the branches taught in the College. Honorable mention was made of Charles Lowndes Steel, of Virginia.

The Second Prize—A Set of Forceps, from Messrs Snowden and Cowman, of the well known Baltimore Dental Depot, to Myron M. Maine, of Connecticut, for the best operation of filling teeth before a Committee of the Board of Visitors. Honorable mention was made of Edgar R. Rust, of Virginia.

The Third Prize—A Dental Engine from S. S. White, Philadelphia, to William G. Foster,of Maryland, for the best piece of plate work presented to a Committee of the Board of Visitors for examination. Honorable mention was made of Benjamin H. Douglass, of Massachusetts, Benjamin Flannigain, of Maryland, Frank M. Seebold, of Pennsylvania, James V. Calver, of District of Columbia, Edward R. De Normandie, of Pennsylvania and Edwin B. Smith, of Pennsylvania.

The Fourth Prize—A Set of Varney Pluggers, from S. S. White, Philadelphia, to Frank Morris Seebold, of Pennsylvania, for the best essay on, and specimen of, Pivot Teeth, presented for examination to a Committee of the Board of Visitors. Honorabla mention was made of Effingham Wagner, of Alabama.

The Class Valedictory was delivered by B. Holly Smith, Jr. of Maryland, the composition and delivery of which were in the highest degree creditable to the orator, being considerably above the average standard of such addresses.

At the annual meeting of the Alumni Association which was held during the morning of Commencement day, the following officers were elected for the ensuing year; President: Dr. F. J. S. Gorgas, of Maryland; 1st, Vice President: Dr. L. M. Cowardin, of Virginia; 2nd Vice President: Dr. B M. Wilkerson, of Maryland; Treasurer: Dr. T. S. Latimer, of Maryland; Corresponding Secretary: Dr. Thomas E. Craddock, of Virginia; Recording Secretary: Dr. Walter S. Harbin, of District of Columbia. The Alumni Banquet was held at the Eutaw House on Commencement evening. Dr. F. J. S. Gorgas presided. The toasts were: "The Dental Profession of America," responded to by Dr. F. N. Seabury, Rhode Island. "The Dental Profession of Europe," responded to by Dr. Ludwig Brandt, of Germany. "The Medical Profession," responded to by Prof. T. S. Latimer, M. D. of Maryland. "The Baltimore College of Dental Surgery," responded to by Prof. J. B. Hodgkin, of District of Columbia. "The Alumni of the Baltimore College of Dental Surgery," responded to by Dr. J. Ryerson Smith, of South Carolina. "The Board of Visitors of the Baltimore College of Dental Surgery," responded to by Dr. Wm. Farmer, of Virginia. "The Graduating Class of 1881," responded to by Dr. B. Holly Smith, of Maryland. "Artistic Dentistry," responded to by Dr. Coryden Palmer, of New York. "The Dental Profession of the South," responded to by Dr. W. F. Tigner, of Georgia. "The Dental Profession of the North," responded to by Dr. F. A. Levy, of New Jersy. "The Dental Profession of Baltimore City," responded to by Dr. George W. Massamore, of Maryland. "Dental Education," responded to by Prof. R. B. Winder, of Maryland. "Session 1880–81," responded to by Dr. T. E. Craddock, of Virginia. "The Junior Class of 1880-81," responded to by Mr. J. B. Harris, of Virginia. "Dental Inventions," responded to by Dr. B. M. Wilkerson, of Maryland. "Dental Literature," responded to by Prof. F. J. S. Gorgas, of Maryland. The Committee of Arrangements were Drs. C. E. Duck, George W. Massamore and William A. Mills. The banquet was served in excellent style, and every thing passed off very pleasantly.

